# Supplementary material for: Risk tolerance and control perception in a game-theoretic bioeconomic model for small-scale fisheries
Source: R Soc Open Sci. 2020 Jul 22;7(7):200621. doi: 10.1098/rsos.200621 (PMC7428242; doi:10.1098/rsos.200621)
Supplement: Azevedo_ESM [file rsos200621supp1.pdf]

ELECTRONIC SUPPLEMENTARY MATERIAL FOR THE ARTICLE:

Risk tolerance and control perception in a game-theoretic bioeconomic model for small-scale fisheries.

Supplementary Material *S1* – The Payoffs equations.
Supplementary Material *S2* – Manipulating the model.
Supplementary Material S3 – Algorithms (software: R)
Supplementary Material S4 – The System.
Supplementary Material *S5* – Systems parameter's.

Supplementary Material *S1* – The Payoffs equations.

Case 1 – Cooperator meets cooperator

In this case, both individuals are cooperators. For that reason, we can assume that fishing effort for each player is f^*, the maximum effort regulated by law. So, we can calculate how much each of these players will won. If c is the cost of a fishing effort unit for the whole season, the total cost for each fisher will be $Cost = c \cdot f^*$. The revenue for the cooperator is $Revenue = H \cdot P$. Here, H is the harvest and P is the market price of the prey.

Harvest equation tell us that

$$H = B(1 - e^{-q \cdot f})$$

But $f = f^*$, so:

$$H = B(1 - e^{-f^* \cdot q})$$

And then:

$$\pi_{CC} = Revenue - Cost = B\left(1 - e^{-q \cdot f^*}\right) \cdot P - c \cdot f^*$$

Finally,

$$\pi'_{CC} = \delta \cdot \left(B\left(1 - e^{-q \cdot f^*}\right) \cdot P - c \cdot f^*\right)$$

Case 2 – Cooperator meets cheater

Suppose that cooperator meet a cheater. While cooperator is fishing with the fishing effort regulated by law, cheater is using all the effort she/he can to maximize her/his profit, despite of the regulation.

Payoff for the cheater is

$$\pi_{NC} = B(1 - e^{-q \cdot f}) \cdot P - c \cdot f$$

For maximize this equation, lets derivate this function by f and make it zero.

$$\frac{\partial \pi_{NC}}{\partial f} = B(0 - (-q \cdot e^{-q \cdot f_{max}}) \cdot P - c = B \cdot P \cdot q \cdot e^{-q \cdot f_{max}} - c = 0$$

So,

$$e^{-q \cdot f_{max}} = \frac{c}{B \cdot P \cdot q}$$

And then,

$$f_{max} = -\frac{\ln\left(\frac{c}{B \cdot P \cdot q}\right)}{q}$$

With the maximum fishing effort, we can calculate the maximum harvest by

$$H_{max} = B(1 - e^{-q \cdot f_{max}})$$

From that harvest, some part will be for the cooperator and some other part (the bigger part) for the cheater. Harvested biomass will be proportional to each player fishing effort. Then

$$H_C = \frac{f_A}{f_{max}} H_{max}$$

$$H_N = \frac{f_B}{f_{max}} H_{max}$$

But $f_C = f^*$ and $f_C + f_N = f_{max}$. So, $f_N = f_{max} - f^*$. And then, for H_C we have:

$$H_C = \frac{f^*}{f_{max}} H_{max}$$

$$H_C = \frac{f^*}{f_{max}} B(1 - e^{-q \cdot f_{max}})$$

$$H_C = \frac{f^*}{-\frac{\ln\left(\frac{c}{B \cdot P \cdot q}\right)}{q}} B\left(1 - e^{-q \cdot \left(-\frac{\ln\left(\frac{c}{B \cdot P \cdot q}\right)}{q}\right)}\right)$$

$$H_C = -\frac{q \cdot f^*}{\ln\left(\frac{c}{B \cdot P \cdot q}\right)} \cdot B\left(1 - e^{\ln\left(\frac{c}{B \cdot P \cdot q}\right)}\right)$$

$$H_C = -\frac{q \cdot f^*}{\ln\left(\frac{c}{B \cdot P \cdot q}\right)} \cdot B\left(1 - \frac{c}{B \cdot P \cdot q}\right)$$

$$H_C = -\frac{q \cdot f^*}{\ln\left(\frac{c}{B \cdot P \cdot q}\right)} \cdot B\left(\frac{B \cdot P \cdot q - c}{B \cdot P \cdot q}\right)$$

$$H_C = -\frac{f^*}{\ln\left(\frac{c}{B \cdot P \cdot q}\right)} \cdot \left(\frac{B \cdot P \cdot q - c}{P}\right)$$

$$H_C = -\frac{\left(B \cdot q - \frac{c}{P}\right) \cdot f^*}{\ln\left(\frac{c}{B \cdot P \cdot q}\right)}$$

Similarly, for H_N, we have:

$$H_N = \frac{f_N}{f_{max}} H_{max}$$

$$H_N = \frac{f_{max} - f^*}{f_{max}} B\left(1 - e^{-q \cdot f_{max}}\right)$$

$$H_N = \frac{-\frac{\ln\left(\frac{c}{B \cdot P \cdot q}\right)}{q} - f^*}{-\frac{\ln\left(\frac{c}{B \cdot P \cdot q}\right)}{q}} B\left(1 - e^{-q \cdot -\frac{\ln\left(\frac{c}{B \cdot P \cdot q}\right)}{q}}\right)$$

$$H_N = \frac{-\frac{\ln\left(\frac{c}{B \cdot P \cdot q}\right) - q \cdot f^*}{q}}{-\frac{\ln\left(\frac{c}{B \cdot P \cdot q}\right)}{q}} B\left(1 - e^{\ln\left(\frac{c}{B \cdot P \cdot q}\right)}\right)$$

$$H_N = \frac{\ln\left(\frac{c}{B \cdot P \cdot q}\right) - q \cdot f^*}{\ln\left(\frac{c}{B \cdot P \cdot q}\right)} B\left(1 - \frac{c}{B \cdot P \cdot q}\right)$$

Now we can calculate π_{CN} and π_{NC}. First π_{CN}:

$$\pi_{CN} = H_A \cdot P - c \cdot f_A$$

$$\pi_{CN} = -\frac{\left(B \cdot q - \frac{c}{P}\right) \cdot f^*}{\ln\left(\frac{c}{B \cdot P \cdot q}\right)} \cdot P - c \cdot f^*$$

$$\pi_{CN} = -\frac{(B \cdot P \cdot q - c) \cdot f^*}{\ln\left(\frac{c}{B \cdot P \cdot q}\right)} - c \cdot f^*$$

$$\pi_{CN} = \frac{(c - B \cdot P \cdot q) \cdot f^*}{\ln\left(\frac{c}{B \cdot P \cdot q}\right)} - c \cdot f^*$$

And, further, π_{NC}:

$$\pi_{NC} = H_B \cdot P - c \cdot f_B$$

$$\pi_{NC} = \frac{\ln\left(\frac{c}{B \cdot P \cdot q}\right) - q \cdot f^*}{\ln\left(\frac{c}{B \cdot P \cdot q}\right)} B\left(1 - \frac{c}{B \cdot P \cdot q}\right) \cdot P - c \cdot (f_{max} - f^*)$$

$$\pi_{NC} = \frac{\ln\left(\frac{c}{B \cdot P \cdot q}\right) - q \cdot f^*}{\ln\left(\frac{c}{B \cdot P \cdot q}\right)} B\left(1 - \frac{c}{B \cdot P \cdot q}\right) \cdot P - c \cdot \left(-\frac{\ln\left(\frac{c}{B \cdot P \cdot q}\right)}{q} - f^*\right)$$

Adding the tendency to cooperate δ we have:

$$\pi'_{CN} = \delta \cdot \left(\frac{(c - B \cdot P \cdot q) \cdot f^*}{\ln\left(\frac{c}{B \cdot P \cdot q}\right)} - c \cdot f^*\right)$$

and

$$\pi'_{NC} = (1 - \delta) \cdot \left(\frac{\ln\left(\frac{c}{B \cdot P \cdot q}\right) - q \cdot f^*}{\ln\left(\frac{c}{B \cdot P \cdot q}\right)} B\left(1 - \frac{c}{B \cdot P \cdot q}\right) \cdot P - c \cdot \left(-\frac{\ln\left(\frac{c}{B \cdot P \cdot q}\right)}{q} - f^*\right)\right)$$

Case 3 – Cheater meets cheater

In this case both players are cheating upon fishing regulation. We considered, as in [1], zero payoff for both cheaters in this case. When there isn't any regulation above harvest, fishermen act as in an open access fishery and fishes until revenue matches fishery costs. So:

$$\pi'_{A_{NN}} = (1 - \delta) \cdot 0 = 0$$

Supplementary Material *S2* – Manipulating the model.

We manipulate the model in order to get some independency from stock size. To do so we divided all payoffs by constant K, the carrying capacity, introducing, then, two new parameters: $B' = \frac{B}{K}$ and $c' = \frac{c}{K}$.

$$\frac{\pi'_{CC}}{K} = \frac{\delta \cdot (B(1 - e^{-18 \cdot q}) \cdot P - c \cdot f^*)}{K}$$

$$\frac{\pi'_{CC}}{K} = \delta \cdot \left(\frac{B}{K}(1 - e^{-18 \cdot q}) \cdot P - \frac{c}{K} \cdot f^*\right)$$

$$\frac{\pi'_{CC}}{K} = \delta \cdot (B' \cdot (1 - e^{-18 \cdot q}) \cdot P - c' \cdot f^*)$$

Similarly,

For payoffs in case 2 we have:

$$\frac{\pi'_{CN}}{K} = \frac{\delta \cdot \left(\dfrac{18 \cdot (c - B \cdot P \cdot q)}{\ln\left(\dfrac{c}{B \cdot P \cdot q}\right)} - c \cdot f^*\right)}{K}$$

$$\frac{\pi'_{CN}}{K} = \delta \cdot \left(\frac{f^* \cdot \dfrac{(c - B \cdot P \cdot q)}{K}}{\ln\left(\dfrac{c}{B \cdot P \cdot q}\right)} - \frac{c}{K} \cdot f^*\right)$$

$$\frac{\pi'_{CN}}{K} = \delta \cdot \left(\frac{\left(\dfrac{c}{K} - \dfrac{B}{K} \cdot P \cdot q\right) \cdot f^*}{\ln\left(\dfrac{c}{B \cdot \dfrac{K}{K} \cdot P \cdot q}\right)} - c' \cdot f^*\right)$$

$$\frac{\pi'_{CN}}{K} = \delta \cdot \left(\frac{(c' - B' \cdot P \cdot q) \cdot f^*}{\ln\left(\dfrac{c}{B' \cdot K \cdot P \cdot q}\right)} - c' \cdot f^*\right)$$

$$\frac{\pi'_{CN}}{K} = \delta \cdot \left(\frac{(c' - B' \cdot P \cdot q) \cdot f^*}{\ln\left(\dfrac{c'}{B' \cdot P \cdot q}\right)} - c' \cdot f^*\right)$$

For the cheater in the same case we have:

$$\frac{\pi'_{NC}}{K} = \frac{(1-\delta)\cdot\left(\frac{\ln\left(\frac{c}{B\cdot P\cdot q}\right)-q\cdot f^{*}}{\ln\left(\frac{c}{B\cdot P\cdot q}\right)} B\left(1-\frac{c}{B\cdot P\cdot q}\right)\cdot P - c\cdot\left(-\frac{\ln\left(\frac{c}{B\cdot P\cdot q}\right)}{q}-f^{*}\right)\right)}{K}$$

$$\frac{\pi'_{NC}}{K} = (1-\delta)\cdot\left(\frac{\ln\left(\frac{c}{B\cdot P\cdot q}\right)-q\cdot f^{*}}{\ln\left(\frac{c}{B\cdot P\cdot q}\right)}\cdot\frac{B}{K}\left(1-\frac{c}{B\cdot P\cdot q}\right)\cdot P - \frac{c}{K}\cdot\left(-\frac{\ln\left(\frac{c}{B\cdot P\cdot q}\right)}{q}-f^{*}\right)\right)$$

$$\frac{\pi'_{NC}}{K} = (1-\delta)\cdot\left(\frac{\ln\left(\frac{c}{B\cdot\frac{K}{K}\cdot P\cdot q}\right)-q\cdot f^{*}}{\ln\left(\frac{c}{B\cdot\frac{K}{K}\cdot P\cdot q}\right)}\cdot B'\cdot\left(1-\frac{c}{B\cdot\frac{K}{K}\cdot P\cdot q}\right)\cdot P - c'\cdot\left(-\frac{\ln\left(\frac{c}{B\cdot\frac{K}{K}\cdot P\cdot q}\right)}{q}-f^{*}\right)\right)$$

$$\frac{\pi'_{NC}}{K} = (1-\delta)\cdot\left(\frac{\ln\left(\frac{c'}{B'\cdot P\cdot q}\right)-q\cdot f^{*}}{\ln\left(\frac{c'}{B'\cdot P\cdot q}\right)}\cdot B'\cdot\left(1-\frac{c'}{B'\cdot P\cdot q}\right)\cdot P + c'\cdot\left(\frac{\ln\left(\frac{c'}{B'\cdot P\cdot q}\right)}{q}+f^{*}\right)\right)$$

For case 3, not so much work to do:

$$\frac{\pi'_{NN}}{K} = \frac{0}{K} = 0$$

Note that $B' = \frac{B}{K}$ measures the relative stock size. Then, even if we don't know the real stock size, we can work with overfishing, underfishing and other kinds of scenarios.

Supplementary Material S3 – Algorithms (software: R)

Algorithm 1

```
#################################################################################
#################################################################################
##                                  ##
##   Strategic game for restricted fishing effort small-scale fisheries     ##
##                                  ##
##   Author: Eric Zettermann Dias de Azevedo                 ##
##   Date of creation: 16/09/2019                    ##
##   Last update: 22/01/2020                      ##
##                                  ##
#################################################################################
#################################################################################

####################### DESCRIPTION#########################################
#                                  #
# This algorithm models restricted fishing effort small-scale fisheries  #
# using game theory. Two fishers decide to cooperate or not to          #
# cooperate with the fishing effort restriction regulation. To cooperate #
# is to follow the restriction and not to cooperate is to fish until     #
# maximize profit.                            #
#                                  #
# This simulations aims to evaluate cooperative behaviour in different   #
# scenarios of control perception and risk tolerance                 #
#                                  #
############################################################################


ls() # List objects
rm(list=ls()) # Remove objects
```

```
if(!require(plot3D)){install.packages("plot3D");library(plot3D)}
if(!require(RColorBrewer)){install.packages("RColorBrewer");library(RColorBrewer)}

####################
#  Initial Set up  #
####################

risk <- 0.3 # risk coefficient
# Represents risk profile of the fisher.
# Values variate from 0 (high-risk toleration fisher) to 1 (low-risk toleration fisher)

alpha <- 1 # Control perception
# Represents how much the fisher feel that the fishery is being controled by autorithies
# Values variate from 0 (fisher did not feel any regulation enforciment) to 1 (fisher
# feel completely controled).


P <- 23 # Market price of the resourse (currency unit)

f <-18 # value of fishing units aloud by restriciotn of the fishing effort

q <- 0.023  # Catchability
# Prepresent how efficient is the fishing gear used to harvest.
# (biomass in kg per fishing unit per season)

cline <- 2*10^(-5) # cost divided per carrying capacity

#####################


#######################
```

```
# Variable Parameters #
#######################

r <- seq(0.01,2,0.01)  # growth rate.

Bline <- seq(0.01,1,0.01) # relative stock size
# represents how is the stock size relative to its carrying capacity.
# B'>> 1 means a stock in its maximum potencial
# B'>> 0 menas a stock too small compared to this potencial to be.

delta <- alpha/(1+risk/r) # tendency to cooperate
# represents how much the fisher is tending to cooperate
# accouting its control perception, risk tolerance and
# the stock growth rate
###################


####################
# Bulding outcomes #
####################

Resultado <- matrix(nrow=100,ncol=200,data=rep(0,2000))
for (i in 1:200){
  for (j in 1:100) {

    Game<- matrix(
      nrow =2,
      ncol=2,
      data=c(delta[i]*(Bline[j]*(1-exp(-f*q))*P-f*cline), # cooperate and cooperate payoff
             (1-delta[i])*((log(cline/(Bline[j]*P*q))-f*q)/log(cline/(Bline[j]*P*q))*Bline[j]*(1-cline/(Bline[j]*P*q))*P+cline*(log(cline/(Bline[j]*P*q))/q+f)), # non-cooparator's payoff when the other cooperates
```

```
        delta[i]*(f*(cline-Bline[j]*P*q)/(log(cline/(Bline[j]*P*q)))-f*cline), #cooparator's payoff when the other not cooperates
        0)
  )

  # Checking for possibles outcomes - Strategies' dominance
  # Using Nowak (2006) criteria.


  output<- ifelse(Bline[j]*P*q<cline,0,                        # no fishing here. Costs are higher than revenue.
          ifelse(Game[1]>Game[2] & Game[3]>Game[4],1,           # domination of cooperation
             ifelse(Game[1]<Game[2] & Game[3]<Game[4],2,        # domination of non-cooperation
                ifelse(Game[1]>Game[2] & Game[3]<Game[4],3,     # biestability
                   ifelse(Game[1]<Game[2] & Game[3]>Game[4],4,5))))) # Coexistence

  #For the coexistence outcome, we determinate cooperative strategy frequency in the equilibrium point (Nowak(2006)).

  output<- ifelse(output==4 & (Game[4]-Game[3])/(Game[1]-Game[3]-Game[2]+Game[4])<0.5,3.5,
          ifelse(output==4 & (Game[4]-Game[3])/(Game[1]-Game[3]-Game[2]+Game[4])>0.5,4,output))

  Resultado[j,i]<-output # creating output matrix.
 }
}
#########################

####################
#   Ploting graph  #
####################

```

```
Resultado[1]<-0 # just to set color scale
Resultado[2]<-4 # from 0 to 4

layout(matrix(c(1,1,2), 3, 1, byrow = TRUE))  # adjust plot window
image2D(t(Resultado), x = seq(0.01,2,0.01), y = seq(0.01,1,0.01),xlab="r",ylab="B'", lighting = F, main = "Game's outputs") #ploting graph
mtext(paste("risco=",risk, " alpha1=",alpha," P=",P," q=",q ," c'=",cline, " f*=",f),side=3) # showing parameters
plot(0, 0, type = "n", bty = "n", xaxt = "n", yaxt = "n",xlab="",ylab="") # adjust for subtitles
legend("topleft", legend=c("no fishing", "domination of cooperation","cooperative coexistence"),fill=c("darkblue", "#0071FF","darkred"),cex=1, bty="n") # subtitles
legend("top", legend=c("domination of non-cooperation", "biestability", "non-cooperative coexistence" ), fill=c("palegreen","orange","red"),cex=1, bty="n") # subtitles

######


################################################
# Evolution of cooperative strategy frequency #
################################################

risk<-0.7
alpha<-1
layout(matrix(1, 1, 1, byrow = TRUE))
r <- seq(0.01,2,0.01)
Blinha<- seq(0.1,1,0.1)
freq <- c(rep(0,200))
plot(freq~r,type="l",lwd=3,col="white",ylim=c(0,1),ylab="Frequency of the cooperative strategy")
cores <- c(brewer.pal(n=9,name='PuRd'),"black") # set color scale

# ploting
for (j in 1:10 ){
```

```
for (i in 1:200){
  delta <- alpha/(1+risk*r[i])  #tendency to cooperate

  PM <-matrix(                # payoff's matrix
    nrow =2,
    ncol=2,
    data=c( delta*(Bline[j]*(1-exp(-f*q))*P-f*cline),
            (1-delta)*((log(cline/(Bline[j]*P*q))-f*q)/log(cline/(Bline[j]*P*q))*Bline[j]*(1-cline/(Bline[j]*P*q))*P+cline*(log(cline/(Bline[j]*P*q))/q+f)),
            delta*(f*(cline-Bline[j]*P*q)/(log(cline/(Bline[j]*P*q)))-f*cline),
            0)
  )


  fC <- (PM[4]-PM[3])/(PM[1]-PM[3]-PM[2]+PM[4]) # frequency of cooperative strategy in equilibrium Nowak(2006)

  fC<-ifelse(fC>1|fC<0,1,fC) # for the case of a domination
  freq[i]<-fC

}
par(new=T)
plot(freq~r,type="l",lwd=3,ylim=c(0,1),col=cores[j],ylab="")
}
mtext(paste("risk=",risk, "control=",alpha," P=",P," q=",q ," c'=",cline, " f*=",f),side=3) # showing parameters
legend("topright",legend=c("B'=0.1","B'=0.2","B'=0.3","B'=0.4","B'=0.5","B'=0.6","B'=0.7","B'=0.8","B'=0.9","B'=1.0"),col=cores,lwd=1) # subtitles for B' values.
```

Algorithm 2

```
####################################################################################
```

```
##############################################################################
##                                                        ##
##   Strategic game for restricted fishing effort small-scale fisheries     ##
##                                                        ##
##   Author: Eric Zettermann Dias de Azevedo                         ##
##   Date of creation: 16/09/2019                              ##
##   Last update: 22/01/2020                                  ##
##                                                        ##
##############################################################################
##############################################################################

###################### DESCRIPTION #####################################
#                                                       #
# This algorithm builds payoff's matrix, generations matrix and a graph  #
# for frequency of the cooperative strategy in a evolutive game using    #
# Replicator's equation (Nowak, 2006)                            #
# scenarios of control perception and risk tolerance                  #
#                                                       #
#########################################################################


ls() # List objects
rm(list=ls()) # Remove objects
if(!require(plot3D)){install.packages("plot3D");library(plot3D)}

##############
# Parameters #
##############

risk <- 0.3
alpha <- 1
```

```
P <- 23
q <- 3*10^(-6)
cline <- 1.92*10^(-5)
Bline <- 0.3
r <- 1

fC <- 0.1 # initial population frequency
fN <- 0.9 #FC + FN = 1


########################
#   Payoff's Matrix   #
########################

delta <- alpha/(1+risk*r)  #tendency to cooperate

MP <-matrix(              #payoff's matrix
     nrow =2,
     ncol=2,
     data=c( delta*(Bline*(1-exp(-18*q))*P-18*cline),
            (1-delta)*((log(cline/(Bline*P*q))-18*q)/log(cline/(Bline*P*q))*Bline*(1-
cline/(Bline*P*q))*P+cline*(log(cline/(Bline*P*q))/q+18)),
            delta*(18*(cline-Bline*P*q)/(log(cline/(Bline*P*q)))-18*cline),
            0)
   )

colnames(MP)<-c("Cooperate","Not cooperate") #names for the columns
rownames(MP)<-c("Cooperate","Not Cooperate") #names for the rows
print("MATRIZ DE PAYOFFS (MP)") #print payoff's matrix
print(MP)                    #on screen

```

```
MP<-MP/max(MP) # adjust matrix values in relation to the bigger value.

############################
# Generation's matrix (G) #
############################

G <- matrix(nrow=101,ncol=2,data=rep(0,202))  # zero's matrix to start
G[1,] <- c(fC,fN) # first row is the initial condition of te frequencies

for (i in 1:100){      #loop for calculate each generation frequencies using replicator's equation (Nowak (2006))

  fit_C = G[i,1]*MP[1,1]+G[i,2]*MP[1,2] # cooperative fitness
  fit_N = G[i,1]*MP[2,1]+G[i,2]*MP[2,2] # non- cooperative fitness
  fit_M = G[i,1]*fit_C+G[i,2]*fit_N     # mean fitness

  var_C = G[i,1]*(fit_C - fit_M) #cooperative frequency variation for the next generation
  var_N = G[i,2]*(fit_N - fit_M) #non- cooperative frequency variation for the next generation

  G[i+1,1]<-G[i,1]+var_C # cooperative frequency for next generation
  G[i+1,2]<-G[i,2]+var_N # non-cooperative frequency for next generation

  k=i+1 #counter

  ifelse(G[i+1,1]>1 |G[i+1,1]<0 ,break,0)  # domination allert
  ifelse(G[i+1,2]>1| G[i+1,2]<0 , break,0) #

  }

G <- G[seq(1,k,1),]              # adjusting matrix
print("Generation Matrix (G)")   # printing
```

```
print(G)


####################
#   Ploting graph  #
####################

par(mfrow=c(1,1))    #ajust window
x <- seq(1,k,1)      # genarations vector
plot(G[,1]~x,type="l",ylim=c(0,1),col="blue",main="Dinamic of the
strategies",ylab="Frequency",xlab="generations",lwd=2) # frequency of coperative strategy for each
genaration
lines(G[,2]~x,col="red",lwd=2) # frequency of non-coperative strategy for each genaration

legend("right", legend=c("coperative", "non-cooperative"), lty=c(1,1), col=c("blue","red"), lwd=2, bty="n")
# subtitles

ifelse(k<100 & G[k,1]>G[k,2],mtext(paste("Cooperative strategy dominates in ",k," generations"),side=3),
# decide between dominance and coexistence and show informations
      ifelse(k<100 & G[k,1]<G[k,2],mtext(paste("Non-cooperative strategy dominates in",k,"
generations"),side=3),
            mtext(paste("Coexistence with: fC= ",round(G[100,1],digits=2)," e fN= ",
round(G[100,2],digits=2)),side=3)))


mtext(paste("risk=",risk, " alpha=",alpha, " P=",P," q=",q," c'=",cline, " B'=",Bline," r=",r," fC=",fC ,"
fN=",fN),side=4) #show parameters
```

Supplementary Material S4 – The System.

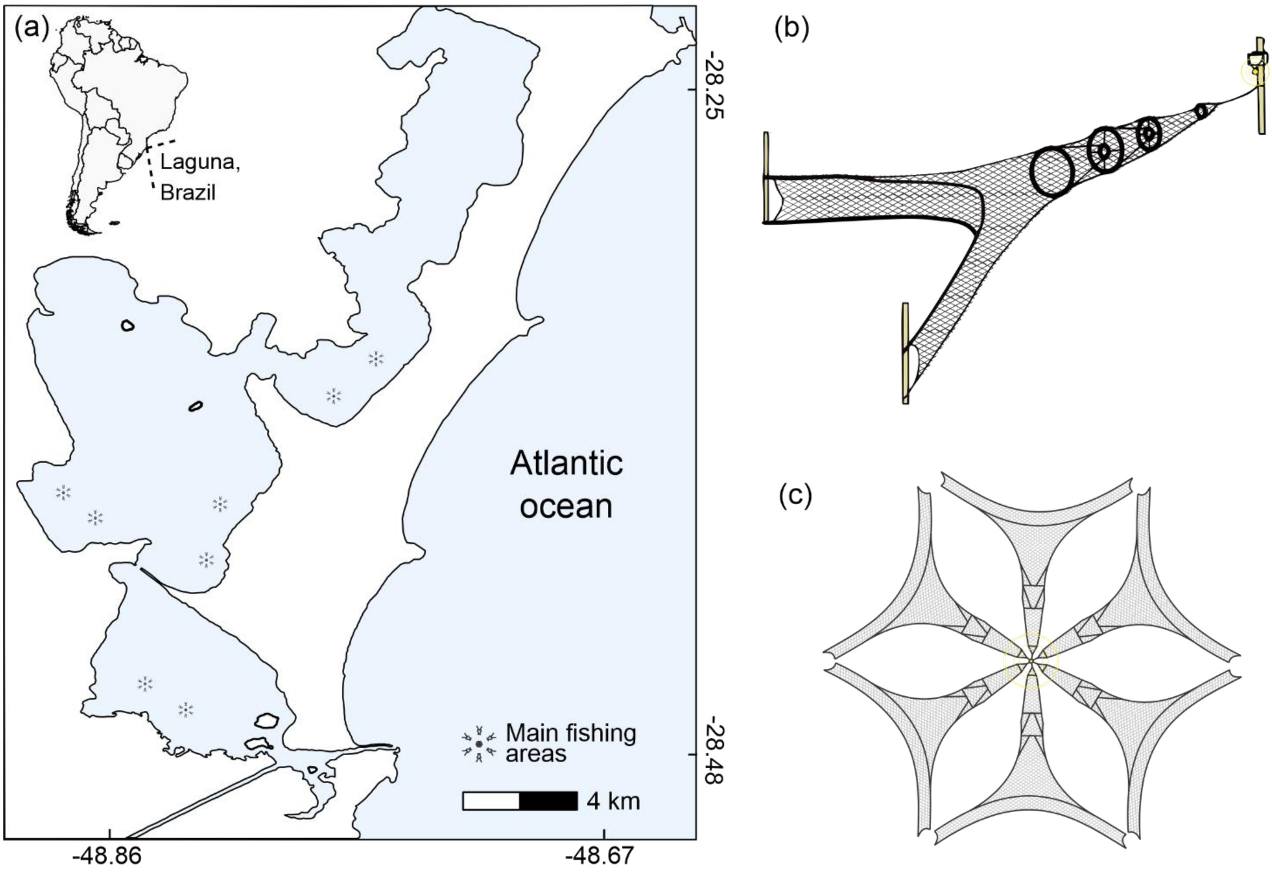

Figure 1. Fishing system characterization. (a) locates the system in the globe showing the main fishing areas while (c) and (d) illustrates the unusual fishing gear used to capture shrimps.

Supplementary Material *S5* – Systems parameter's.

Table 1. Values of case study parameters to test the model.

Inputs	Description	Values	
$\boldsymbol{B'}$	Relative stock size	$0 \leq B' \leq 1$	[1]
$\boldsymbol{r}$	Growth rate	$0 \leq r \leq 2$	[2]
$\boldsymbol{q}$	Catchability	0.023	Empirical
$\boldsymbol{P}$	Fish Market price	23.00	Market price
$\boldsymbol{c'}$	Fishing unit cost/carrying capacity	2×10^{-5}	Empirical
$\boldsymbol{\alpha}$	Fisher's sense of control	$\alpha = 1$ (high fisher's perception of regulation)<br>$\alpha = 0.8$ (low fisher's perception of regulation)	Empirical
$\boldsymbol{b}$	Fisher's risk coefficient	$b = 0.3$ (high fisher's tolerance of risk)<br>$b = 0.7$ (low fisher's tolerance of risk)	[1]

Empirical information and personal investigations, as well as data from technical reports from the case study were used to set the parameters for the model.

The value of B' had to be from zero to on, since it represents relative stock size (stock biomass divided by carrying capacity).

The value of r was taken from zero to two as a range the represents reasonable values for the species [2].

We set the catchability coefficient by $q = 0.023$. We empirically estimated CPUE using technical report data from one fishing season [3]. Then we extrapolated to estimate total stock size using the area of the gear and the area of the lagoons.

The shrimp market price was set in R$ 23,00. This value was researched on-line on May 05th of 2019. We made an arithmetic mean for all values founded.

The $c' = \frac{c}{K}$ coefficient was set by $c' = 2 \times 10^{-5}$. The fishing costs for one season, c was estimated using gas prices, vessels prices, gears prices and information of the logistics of the fishery (e.g. days of fishing and trips per day). We use the extrapolated value of the stock as carrying capacity value, K.

Fishermen's high sense of control was set by $\alpha = 1$. The same parameter was set by $\alpha = 0.8$ for low control perception. Outcomes for the game when the value of this parameter was below 0.8 didn't have any cooperation dominance or cooperative coexistence.

Fishermen's risk tolerance was set by $b = 0.3$ and $b = 0.7$ for high and low tolerance, respectively. This values are the same used in Trisak's model [1].

References

1. Trisak J. 2005 Applying game theory to analyze the influence of biological characteristics on fishers' cooperation in fisheries co-management. *Fish. Res.* **75**, 164–174. (doi:10.1016/j.fishres.2005.03.015)
2. Silva EF, Calazans N, Nolé L, Viana A, Soares R, Peixoto S, Frédou FL. 2015 Population dynamics of the pink shrimp farfantepenaeus subtilis (PÉREZ-FARFANTE ,1967) in northeastern Brazil. *J. Crustac. Biol.* **35**, 132–139. (doi:10.1163/1937240X-00002325)
3. Projeto de Monitoramento da Atividade Pesqueira no Estado de Santa Catarina - UNIVALI. 2018 Informativo estadual Nº 04 (Janeiro a Junho/2018). **02**.
